# Rapid and Accurate Detection of the Most Common Bee Pathogens; *Nosema ceranae*, *Aspergillus flavus*, *Paenibacillus larvae* and Black Queen Cell Virus

**DOI:** 10.3390/insects16080810

**Published:** 2025-08-05

**Authors:** Simona Marianna Sanzani, Raied Abou Kubaa, Badr-Eddine Jabri, Sabri Ala Eddine Zaidat, Rocco Addante, Naouel Admane, Khaled Djelouah

**Affiliations:** 1 Dipartimento di Scienze del Suolo, della Pianta e degli Alimenti, Università degli Studi di Bari Aldo Moro, Via Amendola 165/A, 70126 Bari, Italy; simonamarianna.sanzani@uniba.it (S.M.S.); zaidatsabri@hotmail.com (S.A.E.Z.); rocco.addante@uniba.it (R.A.); 2 Istituto per la Protezione Sostenibile delle Piante, UOS Bari, Consiglio Nazionale delle Ricerche, 701251 Bari, Italy; raboukubaa@ucdavis.edu; 3 Department of Plant Pathology, University of California, Davis, CA 95616, USA; 4 CIHEAM Bari, Via Ceglie 9, Valenzano, 70010 Bari, Italy; badreddinejabri@gmail.com (B.-E.J.); admane@iamb.it (N.A.)

**Keywords:** honey bee, pathogens, qPCR, molecular diagnosis, colony collapse

## Abstract

Honey bees are essential pollinators for the ecosystem and food crops. However, their health and survival face threats from both biotic and abiotic stresses. Fungi, microsporidia, and bacteria might significantly contribute to colony losses. Therefore, rapid and sensitive diagnostic tools are crucial for effective disease management. In this study, molecular assays were developed to quickly and efficiently detect the main honey bee pathogens: *Nosema ceranae*, *Aspergillus flavus*, *Paenibacillus larvae*, and Black queen cell virus. In this context, new primer pairs were designed for use in quantitative Real-time PCR (qPCR) reactions. Various protocols for extracting total nucleic acids from bee tissues were tested, indicating a CTAB-based protocol as the most efficient and cost-effective. Furthermore, excluding the head of the bee from the extraction, better results were obtained in terms of quantity and purity of extracted nucleic acids. These assays showed high specificity and sensitivity, detecting up to 250 fg of *N. ceranae*, 25 fg of *P. larvae*, and 2.5 pg of *A. flavus* DNA, and 5 pg of BQCV cDNA, without interference from bee DNA. These qPCR assays allowed pathogen detection within 3 h and at early stages of infection, supporting timely and efficient management interventions.

## Simple Summary

Honey bee health continues to be increasingly threatened by pathogens such as *Nosema ceranae*, *Aspergillus flavus*, *Paenibacillus larvae*, and Black queen cell virus. This article presents the optimization of specific and sensitive qPCR assays for the rapid detection of these pathogens. New primer pairs to be used in Real-time PCR were designed, and they were combined with a cost-effective CTAB-based procedure for nucleic acid extraction, allowing for the identification of the bee body (abdomen and thorax) as the best portion to be used as sample material. The assays were very sensitive and enabled the detection of pathogens within 3 h, constituting a valuable tool for initial diagnosis and improved management of diseases in honey bee colonies.

## 1. Introduction

Honey bees (*Apis mellifera*) are fundamental to the food industry as they pollinate diverse crops and produce several hive products such as honey and wax. Moreover, honey bees, wild bees, and other pollinators can influence ecosystems by assisting the exchange of genetic materials among angiosperm populations, thus guaranteeing diversity within this widespread plant taxon. However, bee colonies might be affected by several issues, including starvation, queen loss, environmental pollution, climate change, pathogens, and parasites [1]. In particular, since late 2006, a phenomenon called Colony Collapse Disorder (CCD) has been catching the attention of scientists and beekeepers [2,3]. It seems related also to pathogens (viruses, fungi, microsporidia, and bacteria) that might affect honey bee health [4,5].

American foulbrood (AFB), caused by the bacterium *Paenibacillus larvae*, is a global brood disease [6]. The pathogen develops in the gut when ingested by the larvae within 72 h after hatching [7]. In many countries, the presence of AFB must be noted by local authorities, and the colonies with clinical signs should be destroyed, resulting in economic losses for beekeepers and considerable efforts from controlling authorities.

Stonebrood, mainly caused by the fungus *Aspergillus flavus*, has been considered of minor importance for honey bee colonies. However, its incidence is growing reasonably because of climatic changes [8]. In stonebrood, dead bodies of larvae become hard as stone and whitish-gray to black. Furthermore, *A. flavus* produces aflatoxins, which can eventually be found in honey [9].

The microsporidium *Nosema ceranae* is one of the most important adult honey bee pathogens, transmitted mainly via the fecal–oral route. It is spread by transferring the feces of diseased individuals to uninfected ones via ingestion. Within a few weeks after the initial infection, millions of new spores can be found inside the midgut that, once excreted with feces, become new infection sources [10]. Nosema infection causes digestive disorders, shortens life spans, decreases colony size, and reduces honey production [11,12].

However, the largest class of honey bee-infecting pathogens is positive-sense single-stranded RNA viruses, including the Black Queen Cell Virus (BQCV) [5]. Honey bee viruses may cause nonspecific symptoms such as deformities, paralysis, or death. They are transmitted vertically and horizontally among co-feeding wild and honey bee populations, although the mite *Varroa destructor* plays a major role [13,14].

As the conventional diagnosis of pathogen presence is mainly related to the observations of pathogens’ signs, when few or no contrasting measures are available or to prevent the pathogens from infecting a new brood, early diagnosis might be of paramount importance to contribute to reducing disease spreading [15,16,17,18,19]. Furthermore, diagnostic tools could play a major role in the establishment of effective control strategies. In this regard, despite some molecular tools already being available based on conventional PCR [7,8,10], some updates in terms of rapidity and sensitivity might be obtained.

As such, the aims of this study were the setup of rapid, sensitive, and efficient molecular assays for selecting relevant honey bee pathogens (a fungus, a bacterium, a virus, and a microsporidium), and their validation.

## 2. Materials and Methods

Approximately twenty adult flying honey bees were randomly sampled by closing the entrances of four hives and collecting the bees immediately upon entry using a plastic bag. No significant accumulation of dead bees was observed in front of the hive entrances. The collected bees were kept in 96% ethanol and transferred to refrigerated conditions at the laboratory, where they were stored at −20 °C for further analysis.

### 2.1. Total Nucleic Acid (TNA) Extraction

Twenty-four honey bee parts were processed separately to select the most efficient total nucleic acid (TNA) extraction protocol. Six combinations (Table 1) of extraction protocols using two different parts of honey bees were set up. Three extraction protocols, one based on guanidinium thiocyanate-phenol-chloroform and two based on cetyl-trimethylammonium bromide (CTAB) buffers (one manual and one automated), were applied to honey bee parts (head and the rest of the body), then compared for their affordability as well as yield and quality of the obtained TNA. This latter parameter was assessed by spectrophotometric readings (Nanodrop, ThermoFisher Scientific, Milan, Italy) and gel electrophoresis (TBE 1×, Promega, Milan, Italy). For each honey bee, the head was separated from the body into two sterile microcentrifuge tubes, and then both samples were crushed using two 5 mm Ø iron balls in a tissue lyser (Mixer Mill MM 400, Retsch, Haan, Germany) two times for 20 s at 25/s. The TNA extracts were stored at −20 °C for further analysis.

### 2.2. Guanidinium Thiocyanate-Phenol-Chloroform (Trizol) Method

Each processed sample was homogenized with 600 μL of a guanidinium thiocyanate-phenol-chloroform (TRIzol, Thermofisher Scientific) in a microcentrifuge tube until soft tissues were completely disrupted. After centrifugation at 5000× *g* for 1 min, 500 μL of the supernatant was transferred to a new tube, where 500 μL of TRIzol and 200 μL of chloroform were added. The mixture was mixed vigorously for 15 s, samples were incubated at room temperature for 3 min, and then centrifuged for 15 min at 4 °C and 14,000× *g*. The upper phase was recovered, an equal volume of isopropanol was added for TNA precipitation, and then the samples were incubated at room temperature for 10 min. All tubes were centrifuged at 14,000× *g* for 10 min at 4 °C. Finally, the pellet was washed with 1 mL of cold 75% ethanol, centrifuged at 15,000× *g* for 15 min at 4 °C, air-dried, and resuspended in 100 μL of RNase-free water.

### 2.3. Manual Cetyl-Rimethylammonium Bromide (CTAB) Protocol

Each processed sample was homogenized in a microcentrifuge tube with 700 µL of a CTAB-based lysis buffer (Qiagen, Hilden, Germany) and 20 μL of proteinase K (Promega). The mixed solution was then incubated for 25 min at 70 °C, with mixing every 10 min. Once the temperature dropped, 700 µL of chloroform was added, and the tubes were vortexed and centrifuged at 14,000× *g* for 15 min at 4 °C. Afterwards, 500 µL of the supernatant was transferred to a new microcentrifuge tube and supplemented with 350 μL of isopropanol for TNA precipitation. The tubes were stored at −20 °C for 2 h. After centrifugation at 14,000× *g* for 15 min at 4 °C, the pellet was washed with 1 mL of cold 70% ethanol and centrifuged at 14,000× *g* for 10 min at 4 °C. The pellet was air-dried and resuspended in 100 μL of free-nuclease sterile water.

### 2.4. Automated CTAB-Based Protocol

An automated robot system (Maxwell^®^ RSC 48, Promega) was used. Each sample was homogenized as reported above with 700 µL of CTAB buffer (Promega) and 20 μL of proteinase K (Promega) in a microcentrifuge tube, vortexed, incubated for 30 min at 60 °C, and centrifuged for 10 min at 14,000× *g*. Then, for each sample, 100 μL of lysis buffer provided with the Maxwell^®^ RSC PureFood GMO and Authentication Kit (Promega) was added to 200 μL of bee extract into a well of a Maxwell^®^ RSC cartridge. All homogenized samples were processed following the manufacturer’s protocol with a run time of 40 min.

### 2.5. Primer Design and Specificity Confirmation

To develop a new one-step Reverse Transcriptase quantitative PCR (RT-qPCR) assay for BQCV and Real-time quantitative PCR (qPCR) assays for *N. ceranae*, *A. flavus*, and *P. larvae*, specific primer pairs (Table 2) were designed upon reference sequences available in NCBI Genebank of barcoding genes/regions for each genus, using Primer3 software (https://primer3.ut.ee/ accessed on 25 January 2022) version 4.1.0. At first, the primers were submitted to the online NCBI BLAST (https://blast.ncbi.nlm.nih.gov/Blast.cgi accessed on 27 January 2022) search and blasted against the entire Genbank database to confirm their specificity.

The primers were then tested in qPCR reactions using DNA of reference strains of *N. ceranae* and *P. larvae* provided by Istituto Zooprofilattico Sperimentale delle Venezie (Italy) and of *A. flavus* provided by Dipartimento di Biologia Ambientale, Università La Sapienza di Roma (Italy). Whereas the reference BQCV positive sample was certified by an RT-PCR assay [20] and sequencing of the related amplicon.

### 2.6. Amplification Conditions, Primers Sensitivity and Validation

The qPCR reactions were carried out in 96-well plates in a CFX96 Touch Real-time PCR Detection System (Bio-Rad, Hercules, CA, USA). For BQCV, the amplification mix consisted of 1 × POWERup SYBR green (ThermoFisher Scientific), 0.2 μM of each forward and reverse primer (Table 2), 30 U of M-MLV (Promega), and 50 ng of TNA in a final volume of 11 μL. Cycling parameters were 20 min at 37 °C, 10 min at 70 °C, and 2 min at 95 °C, followed by 40 cycles at 95 °C for 20 s and 58 °C for 20 s. Concerning the other pathogens (*N. ceranae*, *A. flavus*, and *P. larvae*), the reaction mix contained 1 × POWERup SYBR green (ThermoFisher Scientific), 0.2 μM of each forward and reverse primers (Table 2), and 25 ng of TNA in a final volume of 20 μL. Thermal cycling conditions were 2 min at 50 °C, 2 min at 95 °C, and 40 cycles of 95 °C for 15 s and 60 °C for 1 min. The specificity of all the primer pairs was confirmed by melting curve analysis: initial denaturation for 5 min at 95 °C, cooling to 55 °C, and melting from 55 °C to 95 °C with a 0.5 °C transition rate every 10 s. Moreover, to further confirm the amplification of single PCR bands of the expected size, an aliquot (10 µL) of products from each primer pair was subjected to 1.2% agarose gel electrophoresis (TBE 1×, Promega).

Absolute quantification of pathogen DNA/RNA was achieved by constructing standard curves. Target DNA/RNA was serially diluted with sterile water to yield final concentrations ranging from 25 ng/µL to 25 fg/µL for DNA and from 14 ng/µL to 1.4 pg/µL for cDNA, then amplified in triplicate as described above; in negative control reactions, water replaced the nucleic acid. A standard curve was generated by plotting the nucleic acid amounts [log (ng)] against the corresponding Cq value. The curve equation, determination coefficient, and PCR efficiency were calculated using the CFX-associated software. The quantity of DNA was used to calculate the related number of cells/conidia based on specific genome sizes (http://zbi.ee/fungal-genomesize/ accessed on 21 June 2022).

The assays set up herein were further validated by analyzing TNA samples from honey bees collected from local apiaries, spiked in triplicate with the DNA/RNA of the target pathogens at the dilutions mentioned above, and used for building up the calibration curves. The same sample was spiked with the four target pathogens to further test the specificity of the reaction.

### 2.7. Statistical Analysis

Statistical analyses were carried out by R software version 3.6.2, using the Kruskal–Wallis non-parametric test and the post hoc Dunn test.

## 3. Results

### 3.1. TNA Extraction Method Selection

**Figure 1:**
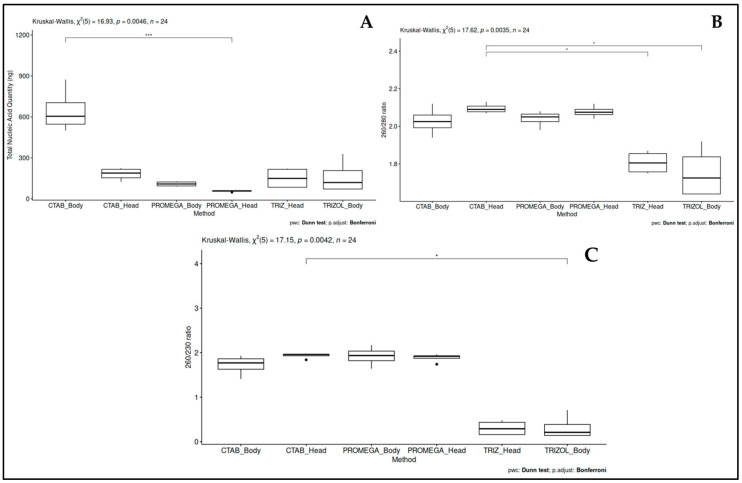
Box plots showing total nucleic acid extraction methods with (**A**) quantity [ng], (**B**) purity based on the 260/280 ratio, and (**C**) purity based on the 260/230 ratio. Each value represents the mean of all replicates ± standard deviation. Statistical analyses were performed using the Kruskal–Wallis non-parametric test (*p*-value shown on each graph), followed by post hoc Dunn tests for pairwise comparisons, with *p*-values indicated by asterisks: * *p* < 0.05, *** *p* < 0.001.

Concerning the comparison of the six combinations of the three TNA extraction methods and the two insect parts (head and the rest of the body), different elements were taken into consideration: yield, purity, and affordability. The statistical analysis showed a significant clustering (*p* = 0.0046) of the data concerning the yield, with the combination CTAB manual protocol (commercial CTAB-based lysis buffer plus proteinase K)/bee body, which resulted well segregated from the other combinations (Figure 1A).

Concerning the purity of the extracted total nucleic acid calculated by the 260/280 (Figure 1B) and 260/230 ratios (Figure 1C) (indicating contamination by proteins and phenolic compounds, respectively), a significant difference (*p* = 0.0035 and *p* = 0.0042, respectively) was observed among samples, resulting in two homogeneous groups: one including the TRIzol/head and TRIzol/body combinations, and the other one the remaining combinations.

Considering the affordability of the protocol, the manual CTAB-based protocol resulted in more cost-effectiveness as compared to the automated one, not requiring specific equipment and dedicated materials.

Following all those results, the manual CTAB-based extraction protocol was selected to be used in the following assays. Furthermore, the honey bee body (excluding the head) was chosen as the target tissue to be used for the extraction.

### 3.2. Primer Specificity

**Figure 2:**
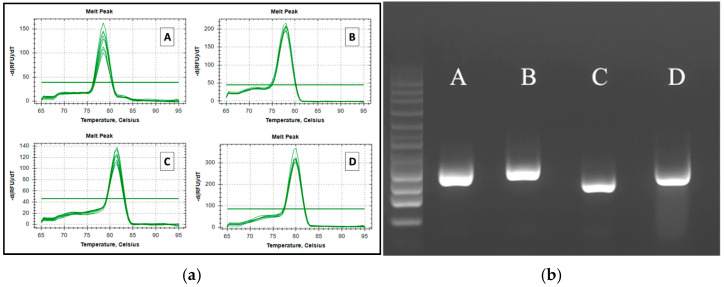
(**a**) Melting curves (left) obtained from qPCR reactions using the newly designed primers for *Nosema ceranae* (**A**), *Paenibacillus larvae* (**B**), *Aspergillus flavus* (**C**), and Black Queen Cell Virus (**D**); (**b**) Related amplicon bands following electrophoresis on agarose gel (right). M: 50 bp ladder (ThermoFisher Scientific).

Initially, primer sequences were run in the BLAST program against the whole Genbank database to test specificity, confirming 100% match only with the target pathogen reference sequences. Furthermore, specific qPCR reactions using positive reference DNA/cDNA templates were run, followed by melting curve analysis and agarose gel electrophoresis. The melting curves resulted in single peaks (Figure 2a), indicating that for each reaction, a single amplicon was generated. Furthermore, running an aliquot of the qPCR amplicons in a 1.2% agarose gel (Figure 2b), single bands of the expected size for each primer pair (Table 2) were observed, confirming the amplification’s specificity.

### 3.3. Assay Sensitivity and Validation

**Figure 3:**
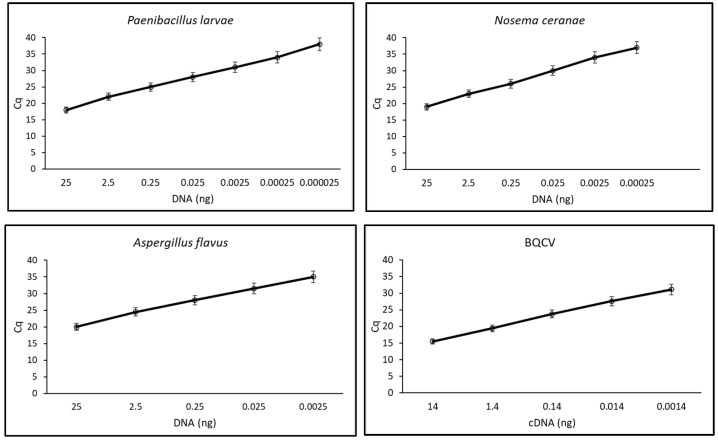
Standard curves obtained amplifying in qPCR reactions serial dilutions of reference pathogen DNA.

Standard curves for the four pathogens were designed, using 10-fold DNA/cDNA dilutions in qPCR reactions (Figure 3). For *N. ceranae*, the standard curve had a slope of −3.560, a y-intercept of 24.373 (R^2^ = 0.998), and an efficiency of 90.9%. The primer pair was able to efficiently amplify up to 250 fg of target DNA (corresponding to 30 cells). For *P. larvae*, the standard curve had a slope of −3.197, a y-intercept of 22.563 (R^2^ = 0.997), and an efficiency of 105.5%. The primer pair efficiently amplified up to 25 fg of target DNA (corresponding to 5 cells). The *A. flavus* standard curve had a slope of −3.406, a y-intercept of 25.970 (R^2^ = 0.999), and an efficiency of 96.6%. The primer pair was able to efficiently amplify up to 2.5 pg of target DNA (corresponding to 66 cells). For BQCV, the standard curve had a slope of −3.370, a y-intercept of 27.711 (R^2^ = 0.982), and an efficiency of 98.0%. The primer pair was able to efficiently amplify up to 5 pg of target cDNA.

Finally, the sensitivity of the assays proved not to be significantly influenced by the co-extracted TNA from the host, revealing comparable standard curves and determination coefficients close to 1.

## 4. Discussion

This article reports the development and validation of molecular assays meant to accurately quantify multiple pathogens (*N. ceranae*, *P. larvae*, *A. flavus*, and BQCV) of honey bees by simplex qPCRs. These assays offer an additional tool for the epidemiological studies of these pathogens, which can easily spread within and among hives. Molecular tools, as compared to conventional cultural methods, allow the rapid and efficient identification of pathogens, i.e., distinguishing between pathogenic and other spore-shaped microorganisms (e.g., yeasts and yeast-like fungi), which might be putative environmental contaminants [21].

Interestingly, the assays proposed herein allowed performing the diagnosis in 3 h. This might be particularly useful, especially for quarantine pathogens, for which rapid decisions must be made. Adult honeybees were selected as targets of the analyses, as they might act as early carriers of pathogens responsible for brood diseases such as American foulbrood and stonebrood, even before the clinical signs appear in the brood itself [22]. These pathogens are easily transmitted by adult bees through feeding and contact within the hive, facilitating the spread of infection [23]. Therefore, sampling adult bees enables early detection and containment of diseases before they severely affect the brood [24].

Furthermore, in the present experiment, the thorax and abdomen of the honey bee were selected as the target body parts, considering that most of the assayed pathogens were reported to concentrate in the abdominal parts of the bee [10]. This could also prevent the co-extraction of eye contaminants, such as pigments, which could inhibit the qPCR reactions [25]. This aligns with a previous study, which reported that the fungal load of *Aspergillus* spp. could vary significantly across body parts and hive compartments, with spore concentrations differing between adult bees and brood or hive materials [26]. In the case of *P. larvae*, our findings supported the recommendations from a previous study [27] and the recommendations of the World Organisation of Animal Health (WOAH) [28], which endorsed the use of honey, hive debris, and adult bees as diagnostic sample types. Those methods involve pathogen detection by conventional PCR with good sensitivity [29,30,31]; however, they require lengthy post-amplification steps. Our qPCR assay achieved a detection limit of 25 fg/μL from adult bees, surpassing the 60 fg/μL threshold previously reported for hive matrices [29], and approaching the detection range of 10 spores/mL using eDNA [30]. Moreover, using asymptomatic adult bees offers a minimally invasive, seasonally flexible, and more immediate diagnostic alternative that can more accurately reflect active infections [23,32].

The sensitivity of these primer pairs proved to be higher than that of other reported assays [8,17,33,34], which might allow the detection even of a few spores/cells, considering the above-reported detection limits and the weight of the genomes of *N. ceranae* and *A. flavus*, which is reported to be of 8 and 36 fg, respectively (www.zbi.ee/fungal-genomesize/ accessed on 21 June 2022).

The design of specific primer pairs for *N. ceranae*, *P. larvae*, *A. flavus*, and BQCV was based on the conserved regions of barcoding genes/regions for the genus [10,19,20,35,36], considering the existing polymorphism in pathogen sequences retrieved from databases, to meet the qPCR reaction requirements. In particular, all the tested primer pairs had an efficiency, where efficiency = [10 (−1/slope)] − 1, included in the optimal range of 90–110% [37] and a R^2^ close to 1.

Using spiked bee homogenates, comparable results were obtained, confirming the accuracy of the method proposed, which largely exceeded the empirically established threshold of 1,000,000 *N. ceranae* spores/honey bee, associated with declining honey bee colonies [11].

The qPCR assay developed for *N. ceranae* successfully detected as little as 250 fg of pathogen DNA, demonstrating high analytical sensitivity and species specificity. This performance aligns with the standards outlined by previous research and the WOAH Terrestrial Manual [25,38], which emphasizes the importance of accurate quantification for epidemiological studies. Moreover, the results support previous findings, which demonstrated the advantages of molecular techniques over traditional diagnosis, especially in identifying low-level infections [39,40].

The real-time PCR detection method for *A. flavus* demonstrated exceptional sensitivity, detecting as little as 2.5 pg of fungal DNA. The assay’s specificity and robustness, unaffected by bee DNA interference, establish it as a promising diagnostic tool. Early molecular detection of fungal pathogens like *A. flavus* is vital because of the often opportunistic and latent nature of the infection [26].

The detection of 25 fg of *P. larvae* DNA is indicative of extremely high sensitivity. The rapid detection time (under 3 h) further enhances the practicality of this assay for field and laboratory use. This is particularly important for *P. larvae*, the causative agent of American foulbrood, which requires immediate action upon confirmation [41]. The findings are consistent with general diagnostic recommendations in previous research [27], which highlight sensitivity and turnaround time as critical performance indicators for molecular tools.

For BQCV, the assay was capable of detecting 5 pg of cDNA, which is consistent with the high sensitivity of molecular diagnostics described in the Standard Methods for Virus Research in *Apis mellifera* [42]. The virus is often asymptomatic during the early stages of infection, making sensitive detection methods vital for preemptive colony health management [43]. Furthermore, a precise diagnosis might be of paramount importance when the diseases are often associated with unclear, chronic, and variable clinical signs.

## 5. Conclusions

The use of the newly developed assays provides a powerful tool for the early, rapid, and sensitive detection of honey bee pathogens (viruses, fungi, microsporidia, and bacteria). These methods can detect a few spores or cells of targeted pathogens at an early stage, potentially identifying even latent or quiescent infections in individuals. Therefore, regular use of these assays can improve disease monitoring, support better management decisions, and lessen reliance on broad-spectrum treatments, fostering more sustainable and precise beekeeping practices. Our findings might open perspectives in the transfer of these qPCR assays to high-throughput methods, as well as their applicability to other bee pollinators.

## Figures and Tables

**Table 1:** Combinations of nucleic acid extraction protocols and honey bee parts used for evaluation.

Protocols	Honey Bee Part
Automated CTAB-method	Head
	Body (excluding the head)
Manual CTAB-method	Head
	Body (excluding the head)
Guanidinium thiocyanate-phenol-chloroform (Trizol)	Head
	Body (excluding the head)

**Table 2:** Primers set up and used in this study.

Pathogen	Primer	Sequence (5′-3′)	Annealing T (°C)	Fragment Size (bp)	Region/Gene (Genbank No.)
*N. ceranae*	NcerRT-F	GCAGCCGCGGTAATACTTGT	60	201	ITS (OL966535)
	NcerRT-F	TCCTGCATTCGACCTCCT			
*A. flavus*	Aflav-F	TAGCCGCCATAATTTTATCCAG	60	127	Calmodulin (OR947226)
	Aflav-R	TTTTGGCCCAGAGAGCGCAT			
*P. larvae*	Plar-F	GGCGACCTTTCAACCCTTGT	60	212	16S rDNA (JF423915)
	Plar-R	TCCTCCGTGTGCTCTTACCA			
BQCV	BQCV1F	GGGAGTCGCAGAGTTCCAAA	58	205	Capsid protein gene (MW442614)
	BQCV1R	CATGAATACAGGGCGGCGTA			

## Data Availability

The data presented in this study are available within the article.
